# Supplementary material for: T-cells produce acidic niches in lymph nodes to suppress their own effector functions
Source: Nat Commun. 2020 Aug 17;11:4113. doi: 10.1038/s41467-020-17756-7 (PMC7431837; doi:10.1038/s41467-020-17756-7)

Supplementary Information

Wu et al., T-cells produce acidic niches in lymph nodes to suppress their own effector functions.

Supplementary Tables S1-S3

Supplementary Figure S1-S18

Supplementary Tables S1-S3

Supplementary Table S1: Flow cytometric count of CD3+ cells in the spleen and inguinal lymph nodes of control mice and of mice treated with anti-CD8 and anti-CD4 antibodies to deplete T-cells.

Mouse	Spleen CD3+ Cells	Lymph Nodes CD3+ Cells
Control M1	34.3	N/A
Control M2	32.1	N/A
Control M3	38.1	58.5
Control M4	29.6	63.5
Control M5	34.8	60.4
Control M6	32.7	74.8
Control M7	35.8	77.5
Control MEAN *(SEM)*	33.91 *(1.04)*	66.94 *(3.87)*
Depleted M7	8.29	1.59
Depleted M8	8.11	15.4
Depleted M9	7.04	17.0
Depleted M10	6.14	13.7
Depleted M11	5.94	11.3
Depleted M12	5.14	17.4
Depleted M13	11.2	14.6
Depleted MEAN *(SEM)*	7.41 *(0.77)*	13.00 *(2.05)*

**Supplementary Table S2**: Estimate of intracellular lactate, based on metabolic rate and pH gradient.							
T-cell	Extra-cellular Ion	pHe	pHi	EACR (relative)	EACR (mM/min)	Lactic acid Perm-eability (µm/s)	[Lactate] (mM)
B6	Cl	6.6	6.916	0.503	5.028	1730	3.302
B6	Cl	6.8	6.995	0.728	7.275	2448	4.051
B6	Cl	7	7.027	1.006	10.057	3572	4.131
B6	Cl	7.2	7.077	1.272	12.718	5319	3.937
B6	Cl	7.4	7.092	1.492	14.921	8004	3.183
OT1	Cl	6.6	6.886	0.326	3.264	1730	2.002
OT1	Cl	6.8	6.947	0.56	5.604	2448	2.795
OT1	Cl	7	6.990	0.864	8.639	3572	3.263
OT1	Cl	7.2	7.062	1.204	12.043	5319	3.603
OT1	Cl	7.4	7.088	1.525	15.248	8004	3.218
B6	Glu	6.6	7.075	0.715	7.147	1730	6.778
B6	Glu	6.8	7.136	1.061	10.61	2448	8.188
B6	Glu	7	7.196	1.259	12.588	3572	7.646
B6	Glu	7.2	7.244	1.562	15.62	5319	7.114
B6	Glu	7.4	7.309	1.857	18.566	8004	6.528
OT1	Glu	6.6	7.078	0.738	7.378	1730	7.049
OT1	Glu	6.8	7.185	1.093	10.929	2448	9.436
OT1	Glu	7	7.225	1.166	11.663	3572	7.572
OT1	Glu	7.2	7.269	1.464	14.643	5319	7.057
OT1	Glu	7.4	7.309	1.746	17.462	8004	6.129

**Supplementary T**able S3. Source and Dilutions of antibodies used for flow cytometry				
Marker	Fluorochrome	Vendor	Clone	Dilution
CD3 /BV421	FITC	BD	145-2cll	1:100
CD4	PacBlu	BD	RM4-5	1:100
CDS	FITC/APC	BD	53-6.7	1:100
CD44	PE	BD	IM7	1:100
CD62L	BUV737	BD	MEL-14	1:100
IFNg	PE	BD	-	1:100
CD19	PE	BD	1D3	1:100
CD40	BV421	BD	3/23	1:100

SUPPLEMENTARY FIGURES AND LEGENDS

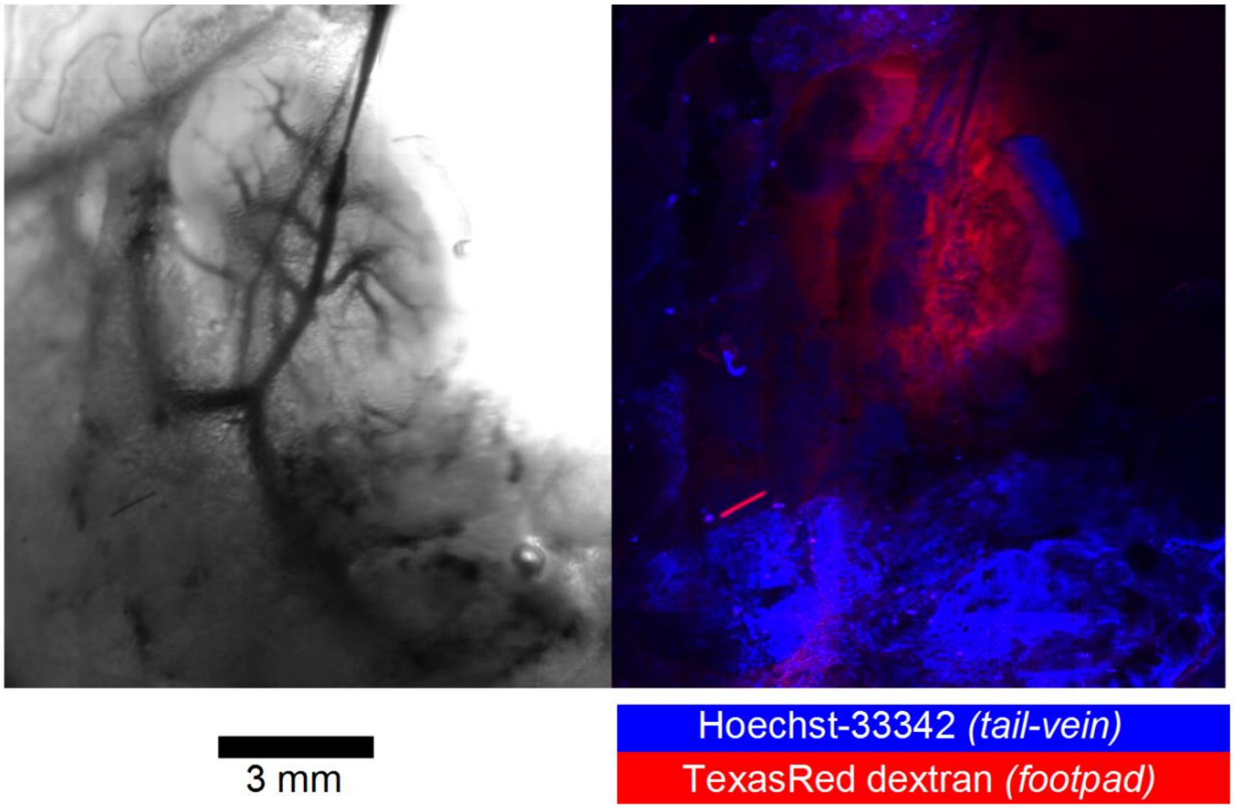

Supplementary Figure S1: *Visualising the LN by intravital imaging using window chambers.* Bright-field and corresponding fluorescence image of LN in control mice injected with Hoechst-33342 into the tail vein (10 mg/kg in 100 µl), to visualize blood vessels, and TexasRed dextran (50mg/ml in 100 µl) into the footpad of the same animal, to demonstrate delivery of substances to the LN. Images collected with x1.6 low power objective. Tail-vein injection occurred 2 hours after footpad injection. Images collected 3 hours after footpad injection. Exemplar of 3 repeats.

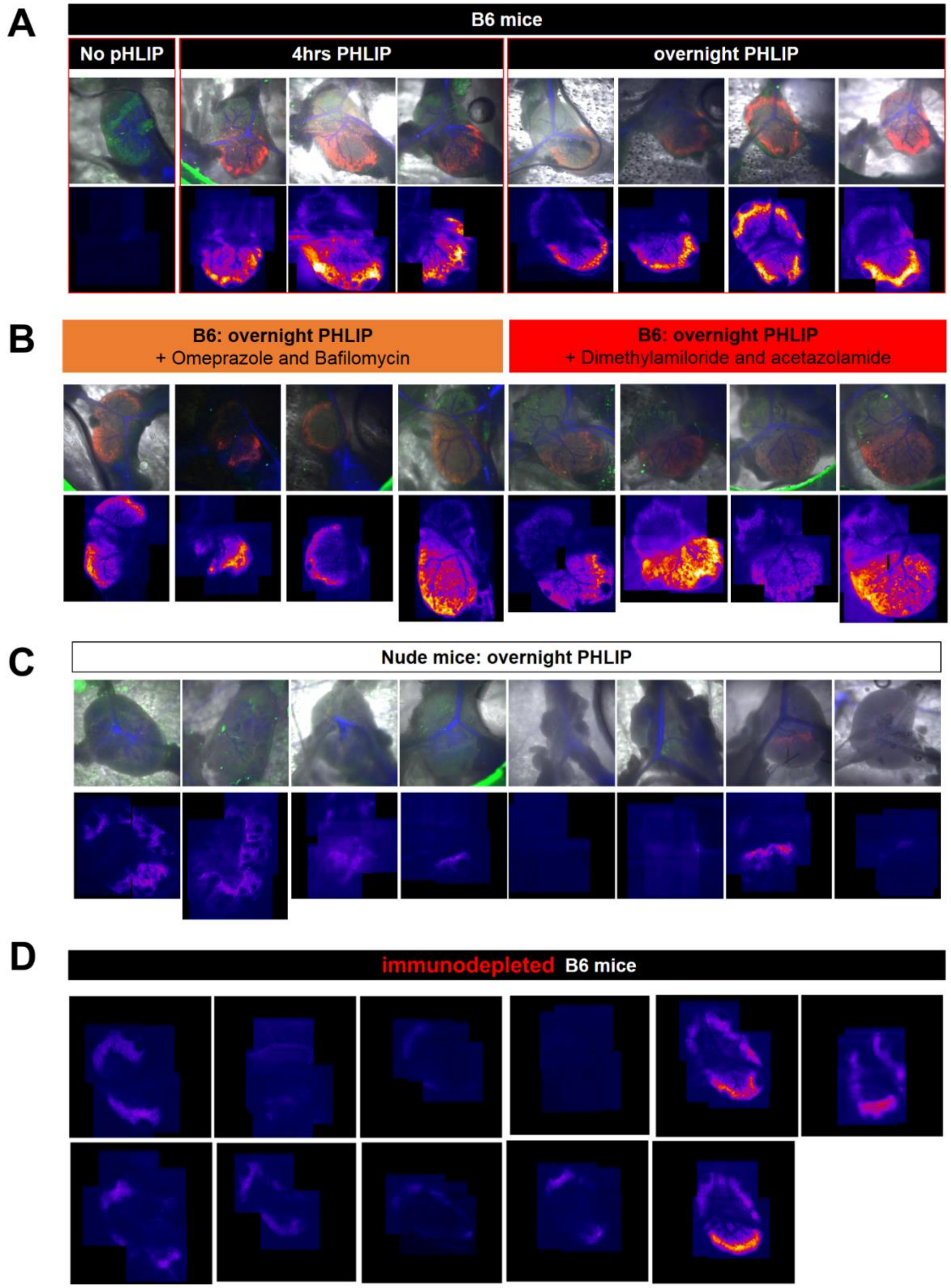

Supplementary Figure S2: *Imaging LNs using pHLIP. Each field of view is 3.8 x 3.8 mm^2.* **(A)** Intravital imaging of LNs of B6 mice for pHLIP. Control experiment had no pHLIP injected, to confirm lack of

background signal. Imaging was repeated on animals with a 50µl footpad injection of 5 nmoles pHLIP either 4 hrs of 24 hrs prior to imaging. Upper panels show superimposition of bright-field image, visualization of blood vessels (blue) and pHLIP fluorescence collected at low power x1.6. Lower panels show montage of overlapping fields of view obtained for pHLIP, collected as a z-stack and summed across the depth. Exemplar Figures from 3, 4 and 10 repeats on different animals. **(B)** Experiments repeated on B6 mice that had received 200µl intraperitoneal injections of 12.5mg/kg of OME and 1mg/kg of BAF or 5.2mg/kg of DMA and 3.9mg/kg of ATZ 24 hours before pHLIP imaging (all data shown). **(C)** Experiments repeated on nude mice that received pHLIP 24 hours before imaging, showing substantial drop in fluorescence (all data shown). **(D)** Experiments on C57/Bl6 mice treated with anti-CD4 and anti-CD8 antibodies (300 µg/mouse) administered IP once per day for 3 days and on every 3rd day thereafter to deplete T-cells (see **Fig S3**). Animals were then injected with pHLIP 24 hours before imaging. (all data shown).

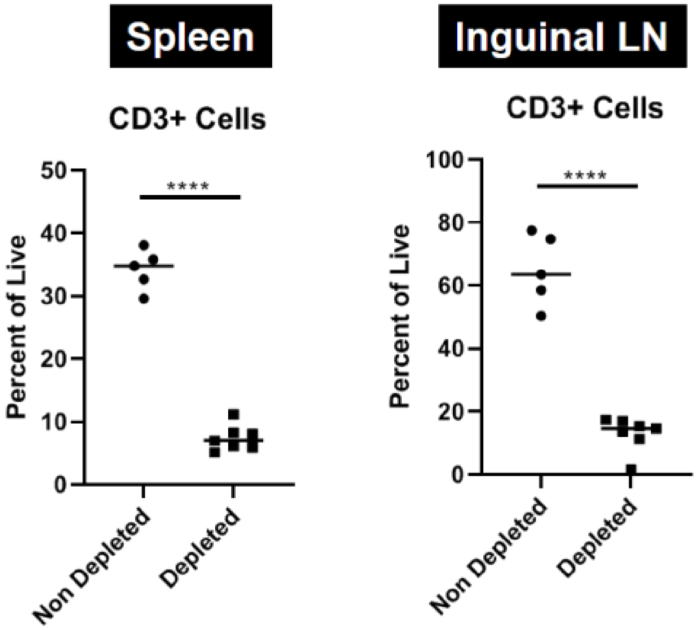

Supplementary Figure S3*: Yield of acute Lymphodepletion.* C57/Bl6 mice (n=7 animals) were lymphodepleted with anti-CD4 and anti-CD8 antibodies (300ug/mouse) administered IP once per day for 3 days and on every 3rd day thereafter. After 10 days, spleens and inguinal lymph nodes were depleted of CD3+ cells, quantified as percent of live cells by flow, and expressed as percent non-depleted (n=5 animals). Two-sided unpaired T-test, $p<0.0001$.

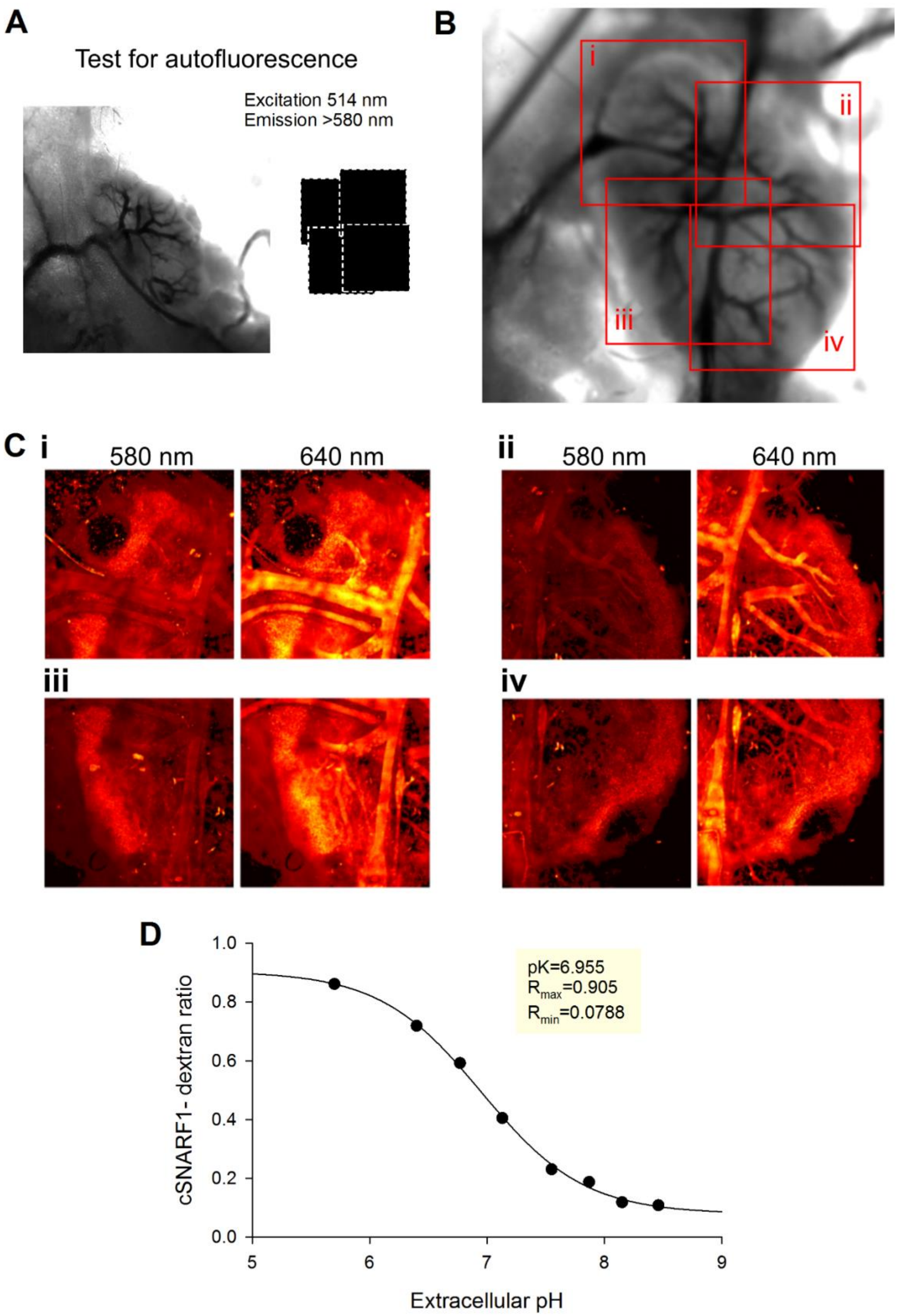
A
Test for autofluorescence
Excitation 514 nm
Emission >580 nm
B
i
ii
iii
iv
C i
580 nm
640 nm
ii
580 nm
640 nm
iii
iv
D
pK=6.955
R max=0.905
R min=0.0788
cSNARF1- dextran ratio
Extracellular pH
1.0
0.8
0.6
0.4
0.2
0.0
5
6
7
8
9

Supplementary Figure S4: *Pipeline for measuring lymph node pH by intravital imaging. Data represent of 18 separate experiments.* **(A)** Bright field image of LN x1.6. Inset shows fluorescence images, collected by x10 objective, of LN region in mice that had not been injected with dextran-cSNARF1. Absence of signal indicates that autofluorescence was negligible under the imaging settings used. *Field of view is 3.8 x 3.8* mm^2 **(B)** Measurements on mouse injected with dextran-cSNARF1. Bright field image of LN at x1.6 where fluorescence was acquired with a higher power (x10) objective. The pHe map of the LN was then constructed by merging the overlapping cascade of images, using anatomical or fluorescence landmarks to align images correctly. *Each box is 1.55 x 1.55* mm^2 **(C)** Pairs of fluorescence at 580 nm (560-600 nm) and 640 nm (620-660 nm) for the four fields of view acquired with x10 objective. *Each box is 1.55 x 1.55* mm^2 **(D)** Calibration curve for cSNARF1-conjugated to 70kDa dextran collected with x10 objective, with best fit to Grinkiewicz equation, showing that the dye is optimal for detecting pH in the range 6-8. *Each field of view is 3.8 x 3.8* mm^2

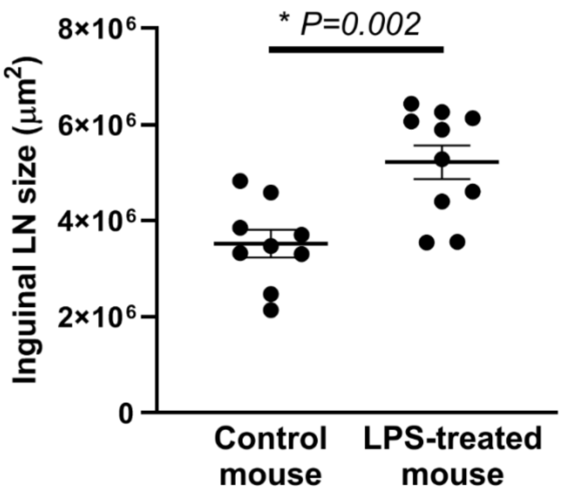

Supplementary Figure S5. *Effect of LPS on LN dimensions.* 100 ng/kg LPS was injected i.p. 48 hours prior to images shown in Fig 1I. In a parallel set of mice inguinal LNs were imaged to measure their volumes (N=10 LNs LPS, N=8 LNs controls, data show mean + SD). The X-Y in plane area increased by 50% (p=0.002), indicating a significant increase in LN volume. Two-sided unpaired T-test.

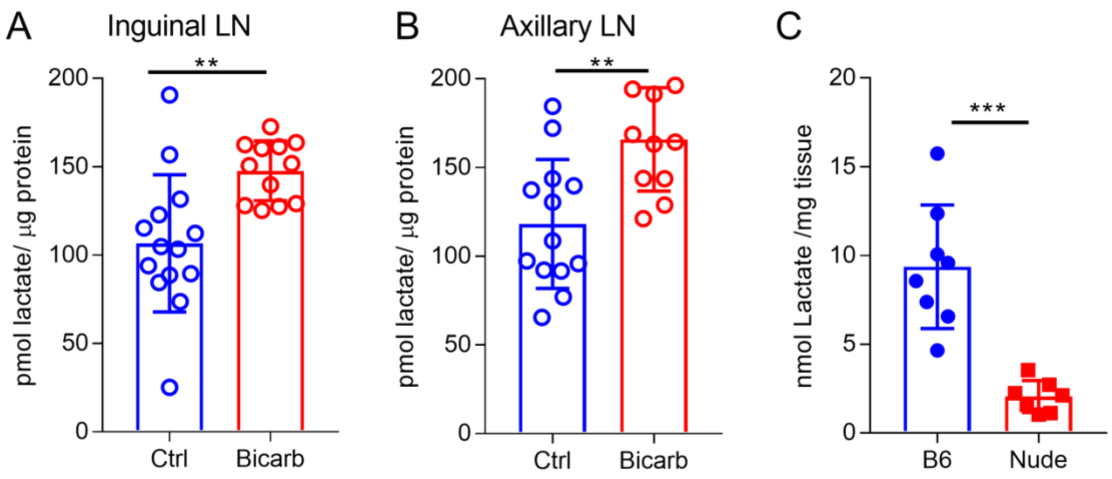

Supplementary Figure S6: *Measurements of [lactate] in LNs.* **(A&B)** Mice were provided oral 400 mM $NaHCO_3$ *ad lib* for one week, after which LNs were removed, weighed and lactate concentrations were measured, normalized to mg protein. Data show significantly more lactate in $NaHCO_3$ treated **(A)** inguinal (control group n=14, bicarbonate group n=12, p=0.0024), and **(B)** axillary (control group n=13, bicarbonate group n=11, p=0.0021) LNs compared to tap water controls. **(C)** Inguinal lymph nodes were removed from C57BL/6 (B6) or nude mice and the lactate concentration were measured. Each lymph node was from separate animal (N=8 each, p=0.00005). Data was expressed as Mean ± SD, and compared using two-tailed, unpaired t-test. Significance level: **p<0.01; ***p<0.001.

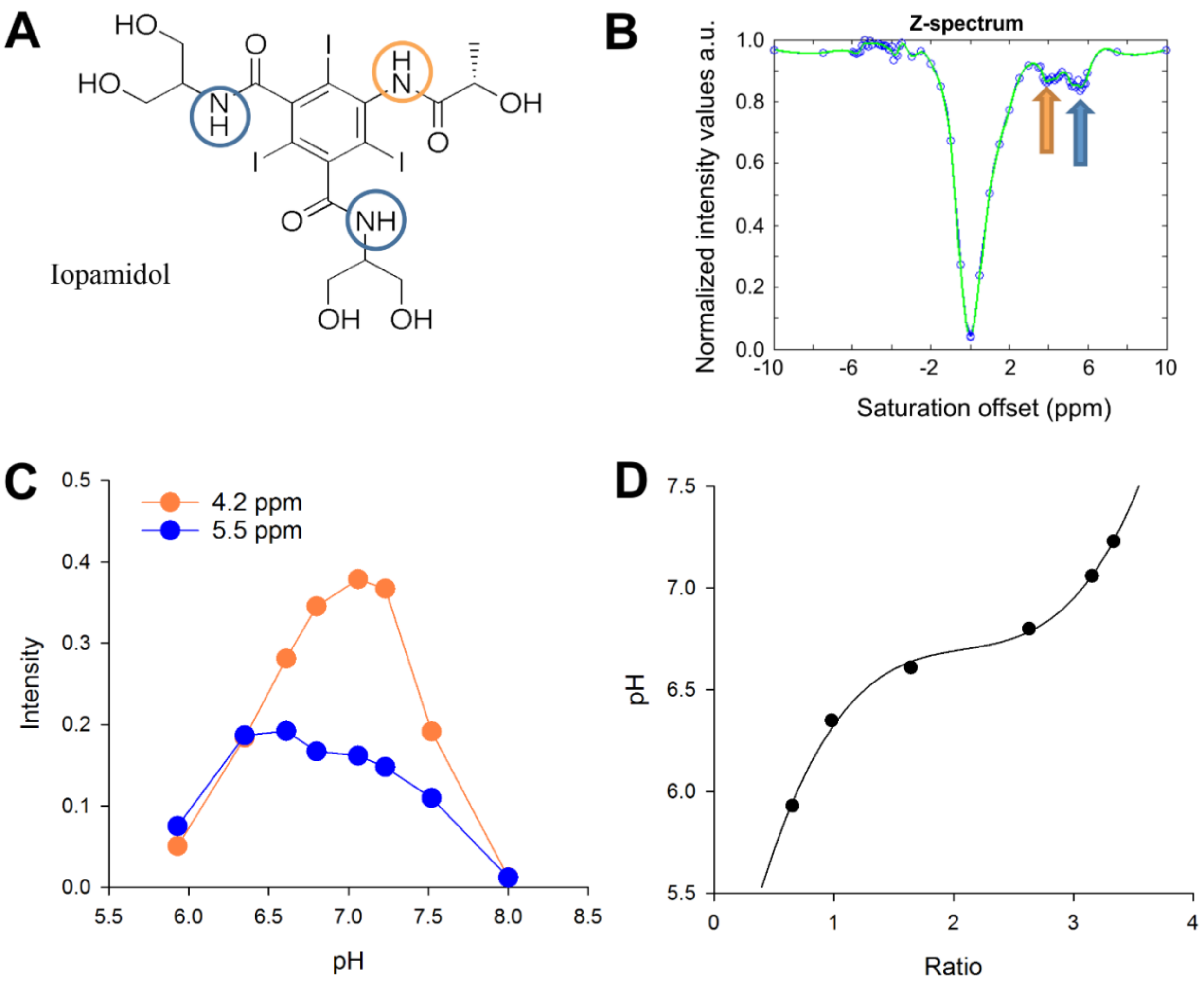

Supplementary Figure S7: *Chemical Exchange Saturation Transfer (CEST) pH imaging.* **(A)** Structure of iopamidol (Isovue) showing ionizable amide groups in red and blue. The two blue groups are co-resonant. **(B)** Z-spectrum generated by measuring water resonance intensity following saturating frequency specific pulses at between +10 and -10 ppm relative to the water resonance at 0 ppm. The two amide resonances are indicated with red and blue arrows. **(C)** Analysis of data from phantoms at different pH. **(D)** Ratio of intensity and its best-fit 5th order polynomial.

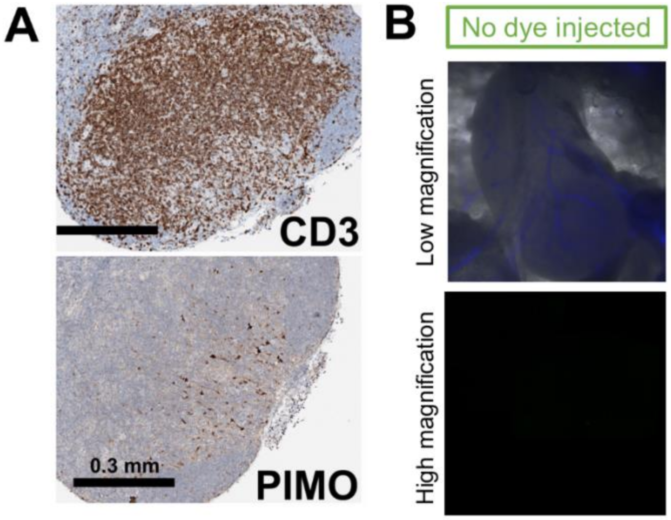

Supplementary Figure S8: *Probing evidence for LN hypoxia.* **(A)** Staining for hypoxia with pimonidazole (Pimo) injected to B6 mice i.p. (60 mg/kg in 100µl) prior to euthanizing. Inguinal LNs were excised for immunohistochemistry. Images are representative of N=10 LN (CD3) and N=3 LN (pimo), plus no primary controls. **(B)** Determination of background signal in intravital imaging of LN in mice that had not received ImageIT-Green at low (FOV = 3.8 x 3.8 mm^2) and high (FOV = 1.55 x 1.55 mm^2) magnification. s

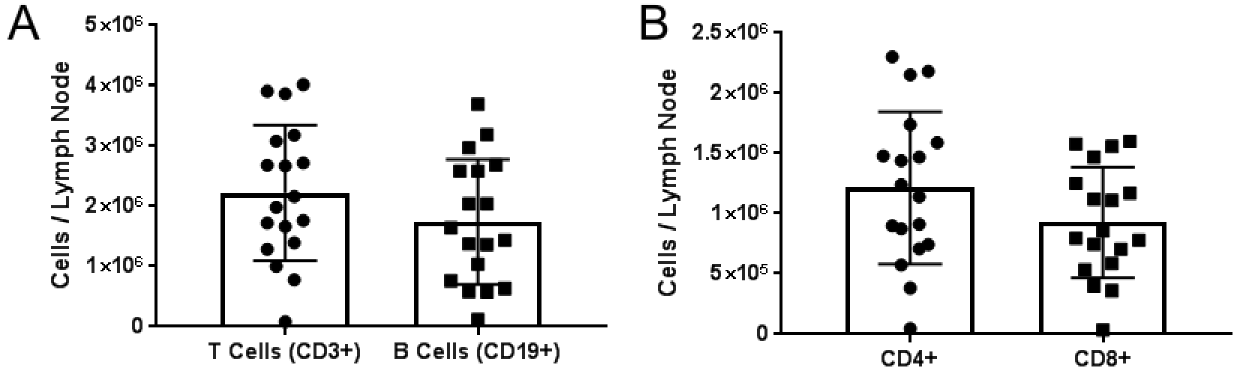

Supplementary Figure S9. *T cell counts in Lymph nodes form B6 and nude mice.* LNs were excised for flow cytometric measurements. **(A)** CD3+ T cells and CD19+ B cells, as well as **(B)** CD4+CD3+ and CD8+CD3+ T cells were quantified by flow (n=18). The volumes of inguinal and axillary LNs of C57Bl6 are around 2.5 ± 0.3 mm^3 indicating that the density of CD3+ cells in LNs is ~800 million per mL. Error bars represent S.D., no statistical test was performed.

FIGURE S6

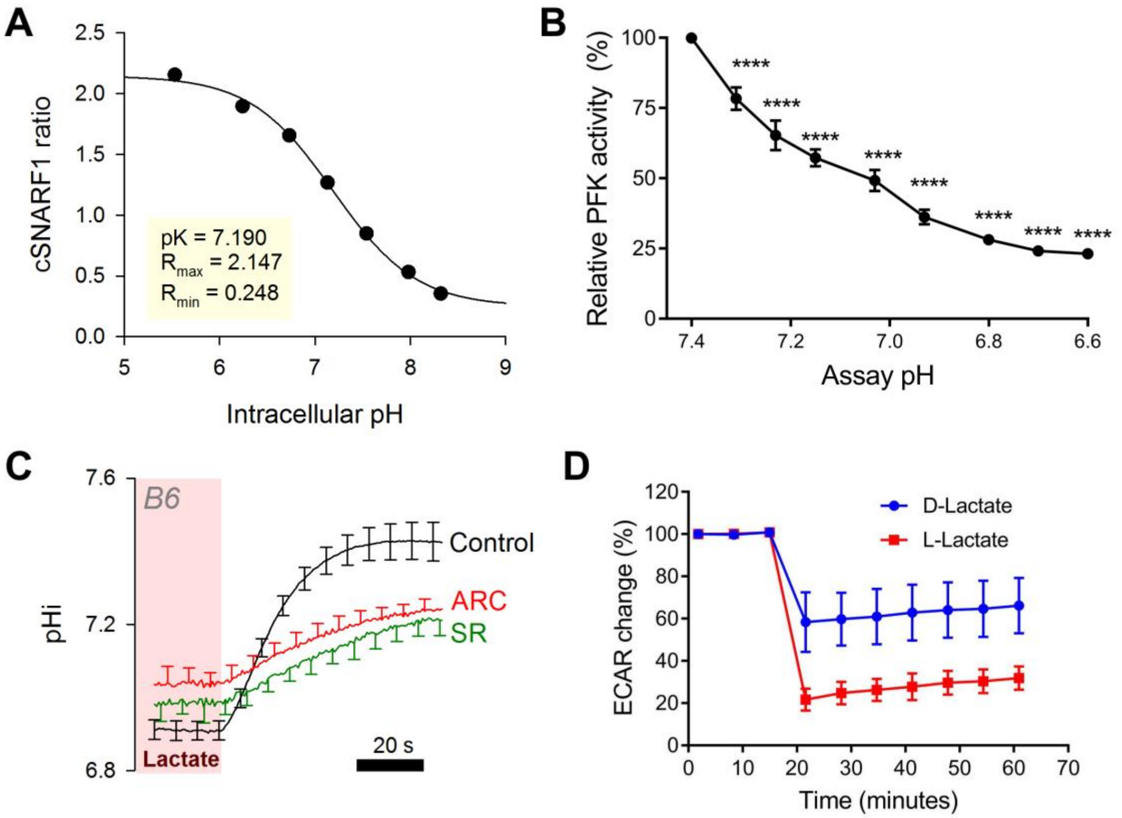

Supplementary Figure S10: *Mechanisms of T cell glycolysis inhibition by acidosis.* **(A)** Calibration curve for cSNARF1 AM-loaded into B6 cells, performed by the nigericin (10 µM) method, showing best fit to Grinkiewicz equation. Mean ± SEM of results from 4 experiments, each with 40-60 cells each. **(B)** PFK-1 enzyme activity of homogenate from activated B6 cells measured at different pH (mean ± SD; n=3;One way ANOVA with multiple comparisons, p=0.0001). **(C)** Effect of MCT1 inhibitors AR-C (AR-C155858; 10 µM; n=7) and SR (SR13800; 10 µM; n=7) on rate of pHi change upon rapid removal of extracellular lactate. The ablated rate arises from inhibited MCT activity. Buffering capacity was assumed to be constant (control; n=12). Mean ± S.E.M. (D) ECAR measured by Seahorse, showing effect of adding L- and D-lactate on glycolytic rate. Note that due to the stereo-specificity of LDH, the inhibitory effect of the L-isoform is stronger (mean ± S.D. ; n=3).

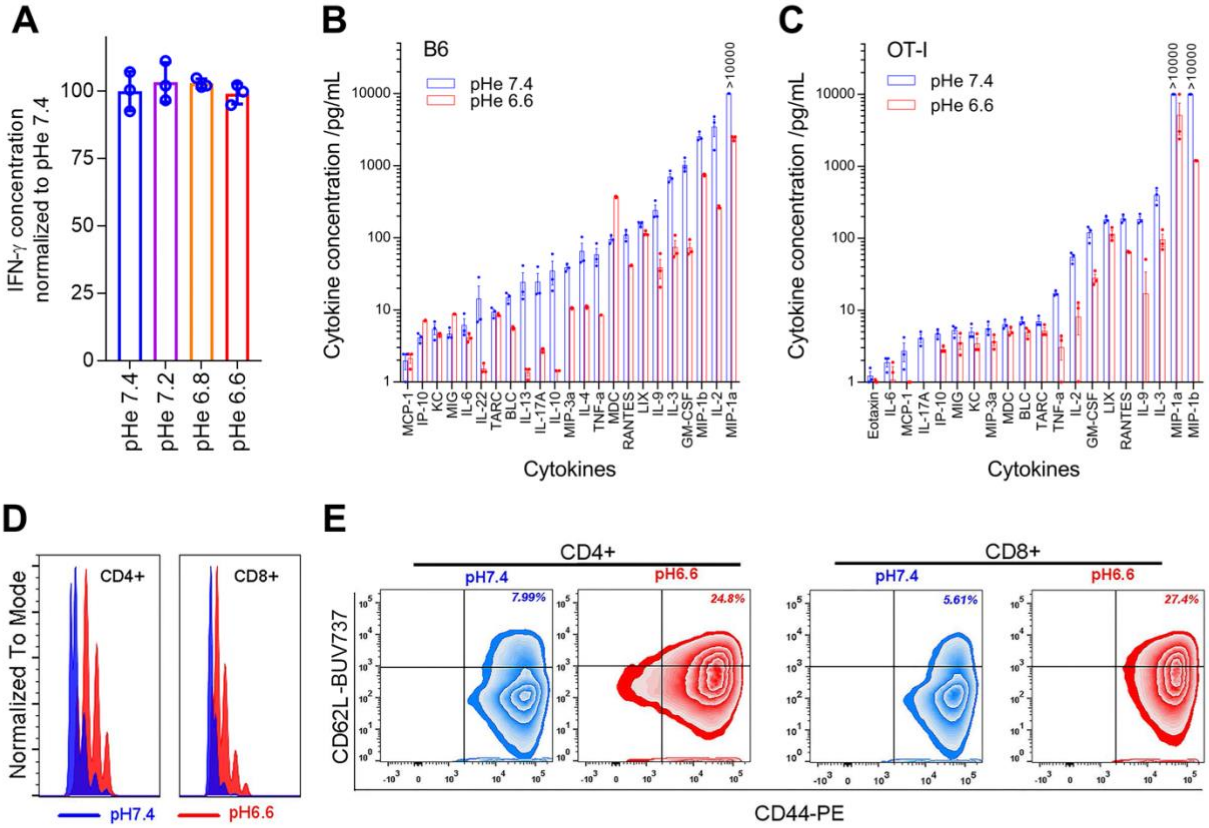

Supplementary Figure S11: *T cells effector functions are inhibited at acidic pH***.** (A) Interferon-γ (IFNγ) immunoreactivity is stable *in vitro* over a range of pHe. Data are showed as Mean ± SD, n=3. **(B)** Relationship between cytokine levels measured in paired experiments at low and high pH by Cytokine Beads Array assay in B6 T cells Data are showed as Mean ± SD, n=3. **(C)** Experiment repeated in OT-I cells. Data shown as Mean ± SD. **(D)** Proliferation rate of $CD4^+$ and $CD8^+$ cell from total B6 T cell at low and high pH. **(E)** Fraction of $CD44^+CD62L^+$ cells (memory phenotype) is increased at low pHe in $CD4^+$ and $CD8^+$ subpopulation from B6 T cell.

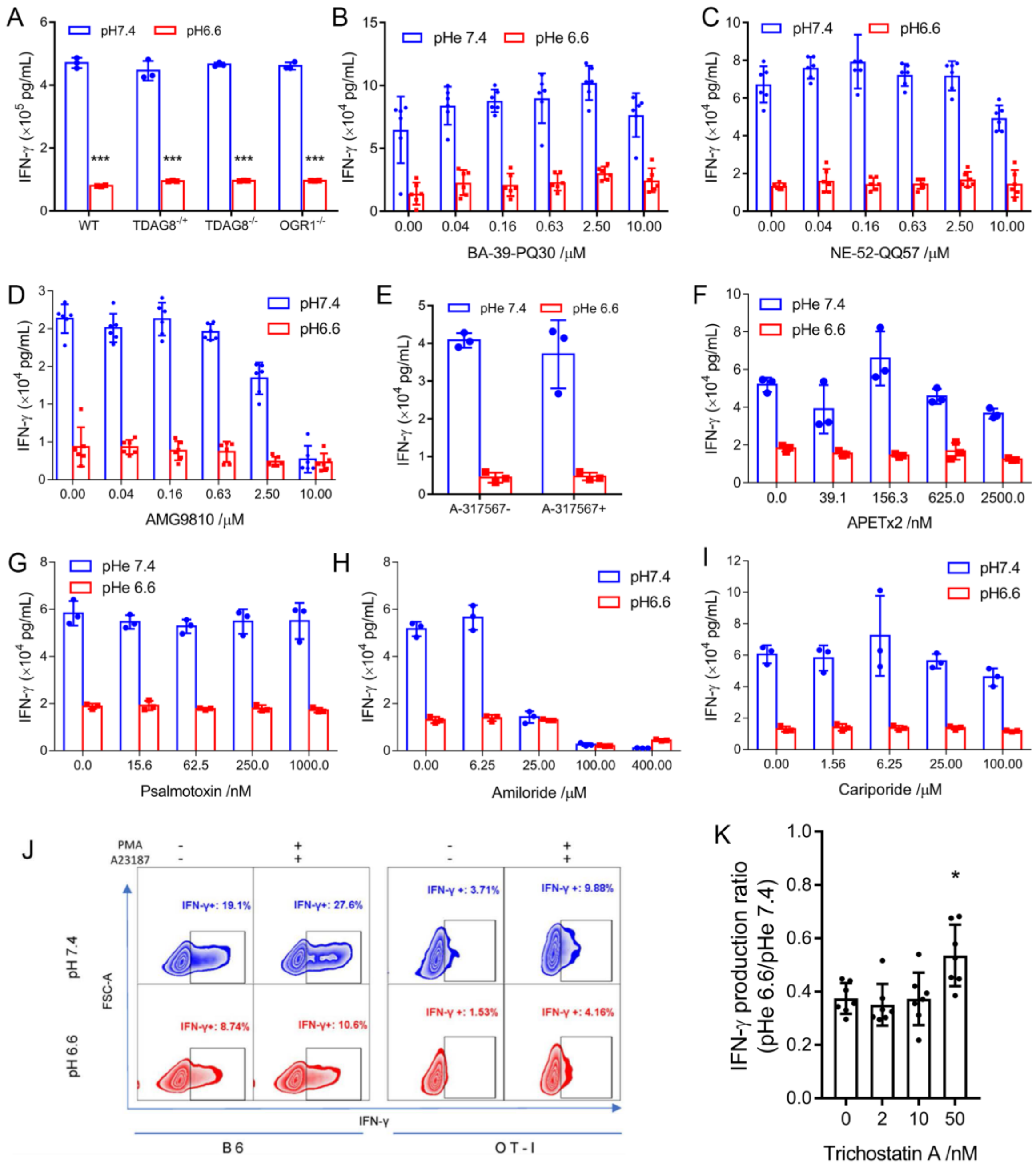

Supplementary Figure S12: *Testing potential mechanisms of pH-sensitivity of cytokine release (all p-values two tailed t-test).* **(A)** G protein-coupled receptor protein TDAG8 homogeneous (TDAG$^{-/-}$) and heterogeneous (TDAG8$^{-/+}$) knockout and OGR1 knockout (OGR$^{-/-}$) showed no rescue of IFNγ production at low pHe (n=3 each; p-values 6.6 cf. 7.4 WT: p=2.08e-6; TDAG8$^{-/+}$: p=4.09e-5; TDAG8$^{-/-}$: p=4.57e-8 ; OGR1$^{-/-}$:p=6.55e-7). **(B)** OGR1 inhibitor BA-39-PQ30 (n=6 each)and **(C)** GPR4 inhibitor NE-52-QQ57 (n=6 each) did not rescue IFNγ production at low pHe. **(D)** Inhibitors of TRPV1 (n=6 each), **(E)** ASIC-3 (n=3 each), **(F)** ASIC-3 (n=3 each), **(G)** ASIC1a (n=3 each), **(H)** ENaC (n=3 each) and **(I)** NHE1 (n=3 each) were unable to rescue IFNγ production at low pHe. **(J)** The phorbol 12-myristate 13-acetate plus A23187 (n=3 each) and

(K) a low dose of Trichostatin A, a histone deacetylase inhibitor, only partially restored IFNγ production at pHe 6.6, compared to pHe 7.4 ((n=7, p=0.0063). Data are expressed as Mean ± SD; Two-tailed, unpaired t test was used for comparison. *, p<0.05; **, p<0.01; ***, p<0.001; ****, p<0.0001.

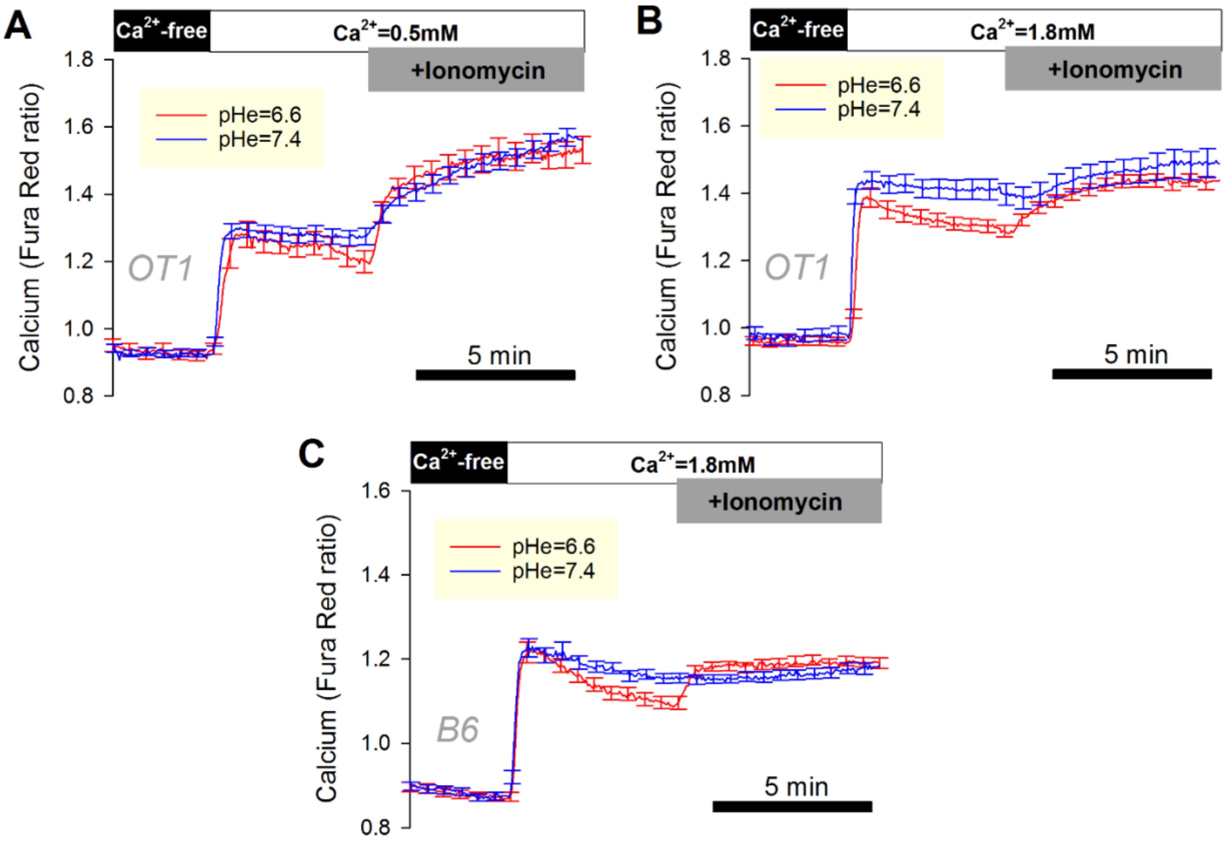

Supplementary Figure S13: *Investigating the effect of acid-inhibition of calcium signaling.* **(A)** Protocol for interrogating store-operated Ca^{2+} entry. Superfused OT-I T cells were loaded with Fura-Red and exposed to 10 µM thapsigargin for 10 min in Ca^{2+}-free (0.5mM EGTA) conditions to deplete the endoplasmic reticulum (ER) Ca^{2+} store. Next, extracellular Ca^{2+} was raised to 0.5 mM by rapid solution switching at either pHe 7.4 or 6.6. The slope and extent of intracellular Ca^{2+} rise (Fura-Red ratio) is a readout of Ca^{2+} entry. Ionomycin (10 µM) was added at the end of the experiment as a positive control for Ca^{2+} entry. N=20 (pH 6.6), 25 (pH 7.4). (B) Experiment repeated with higher Ca^{2+} (1.8 mM) in OT-I cells. N=15 (pH 6.6), 18 (pH 7.4) (C) Experiment repeated with B6 cells using 1.8 mM Ca^{2+}. Time courses show mean ± SEM of 15-25 cells each. N=21 (pH 6.6), 22 (pH 7.4).

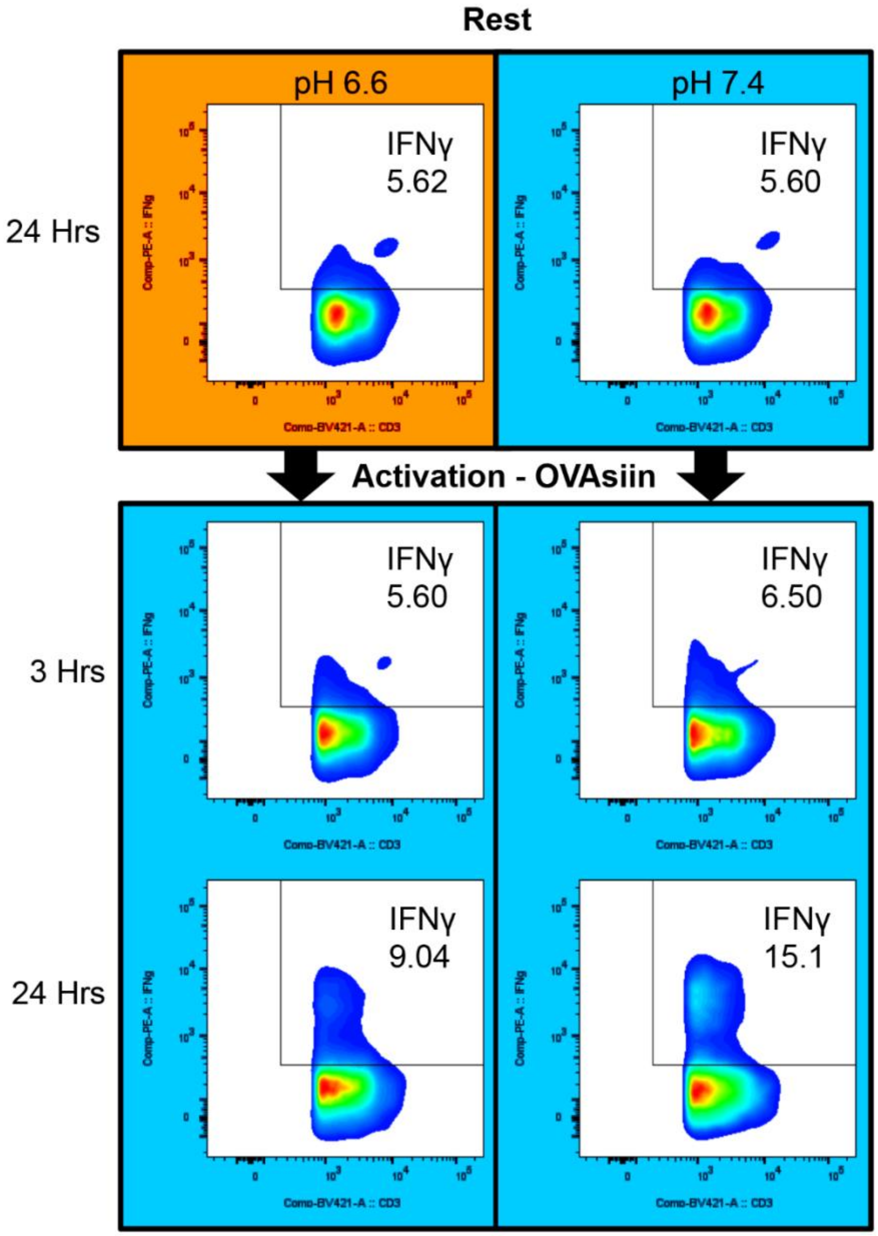

Supplementary Figure S14: *Resting T cells at pHe 6.6 or 7.4 had no effect on IFNγ measured at pHe 7.4 with stimulation*. T cells were rested at pHe 6.6 or 7.4 for 24 hours, and then activated with $OVA_{257\text{-}264}$ (SIINFEKL) peptide at pHe 7.4 for an additional 24 hours. Intracellular IFNγ staining was measured during initial rest period and activation period by flow cytometry. Gating Strategies are shown in Fig. S18A-D.

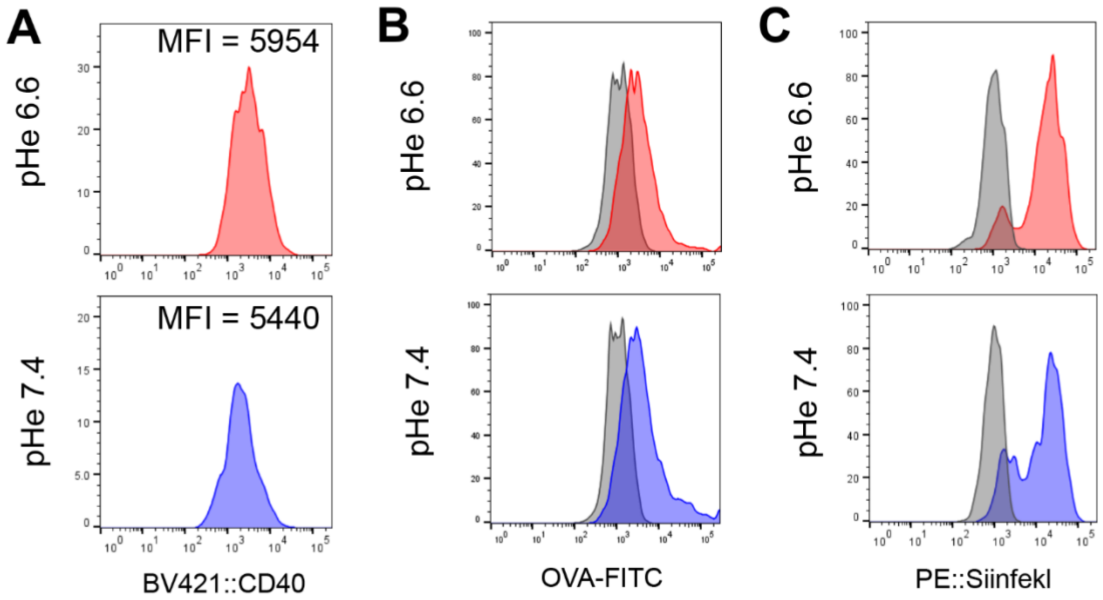

Supplementary Figure S15: *Low pHe showed no effects on dendritic cell (DC) antigen presenting functions.* **(A)** Dendritic cells incubated at pHe 6.6 or 7.4 for 24 hours maintain the same level of CD40 expression. **(B)** Dendritic cells incubated at pHe 6.6 or 7.4 for 24 hours are able to take up FITC-labelled OVA protein. **(C)** Dendritic cells incubated at pHe 6.6 or 7.4 for 24 hours are able to present SIINFEKL peptide.

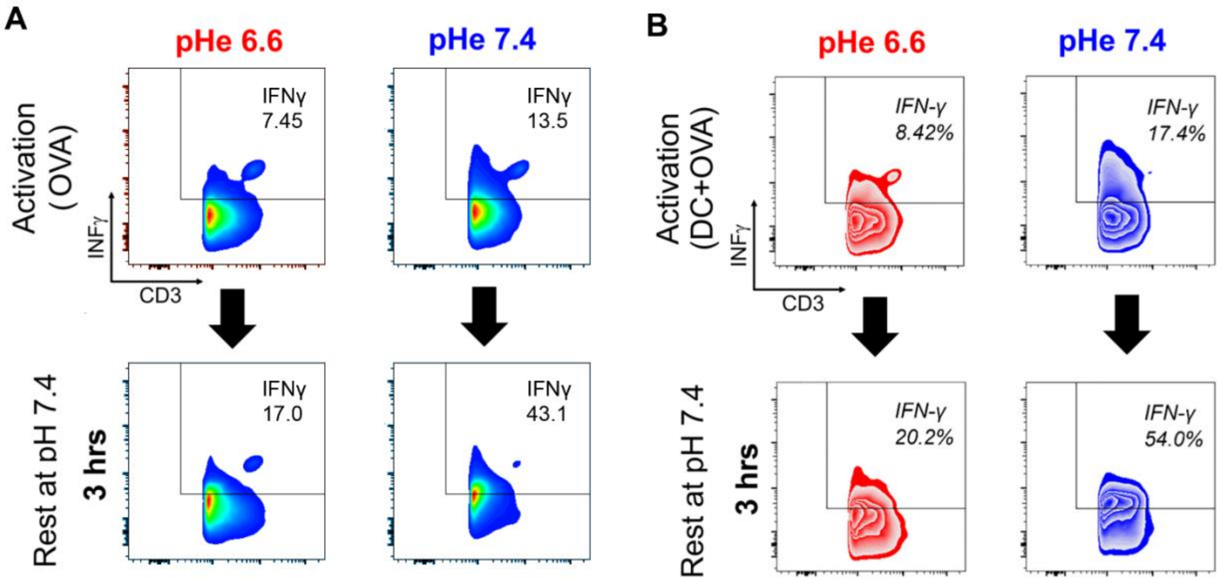

Supplementary Figure S16: *Rescue of low pHe effects on IFNγ production in T cells activated with peptide.* **(A)** Intracellular IFNγ staining of T cells activated at pHe 6.6 or 7.4 for 24 hours with $OVA_{257\text{-}264}$ (SIINFEKL) peptide alone. Cells were then transferred to media at pHe 7.4 and IFNγ production measured by flow at 3 and 24 hours thereafter. **(B)** Experiment repeated with the addition of dendritic cells presenting SIINFEKL peptide during the activation period at pHe 6.6 or 7.4.

A) MCT4
((H90) SC-50329
MW~43 kDa)

B) β Actin
(Sigma A1978
MW- 42kDa)

C) MCT1
(Abcam 90582
MW ~48 kDa)

D) β Actin
(Sigma A1978
MW- 42kDa)

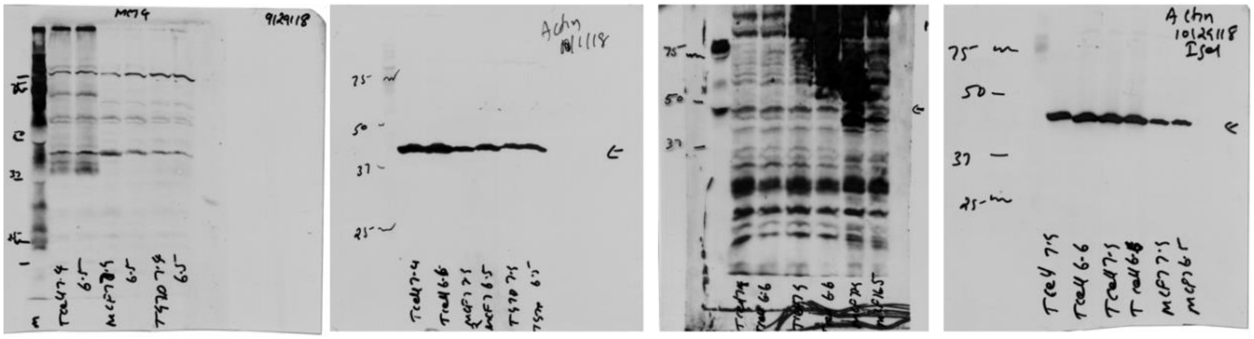

Supplementary Figure S17. Whole blots in support of **Figure 3D** for A) MCT4; B) MCT4 actin control; c) MCT1; D) MCT1 actin control.

Supplementary Figure S18A. Flow gating strategies for cell proliferation and memory phenotype.

Activated T cells was initially gated with SSC-A & FSC-A, then live cells was gated out for doublet cell exclusion. The CD4 and CD8 cells were picked separately for cell proliferation and memory phenotype analysis.

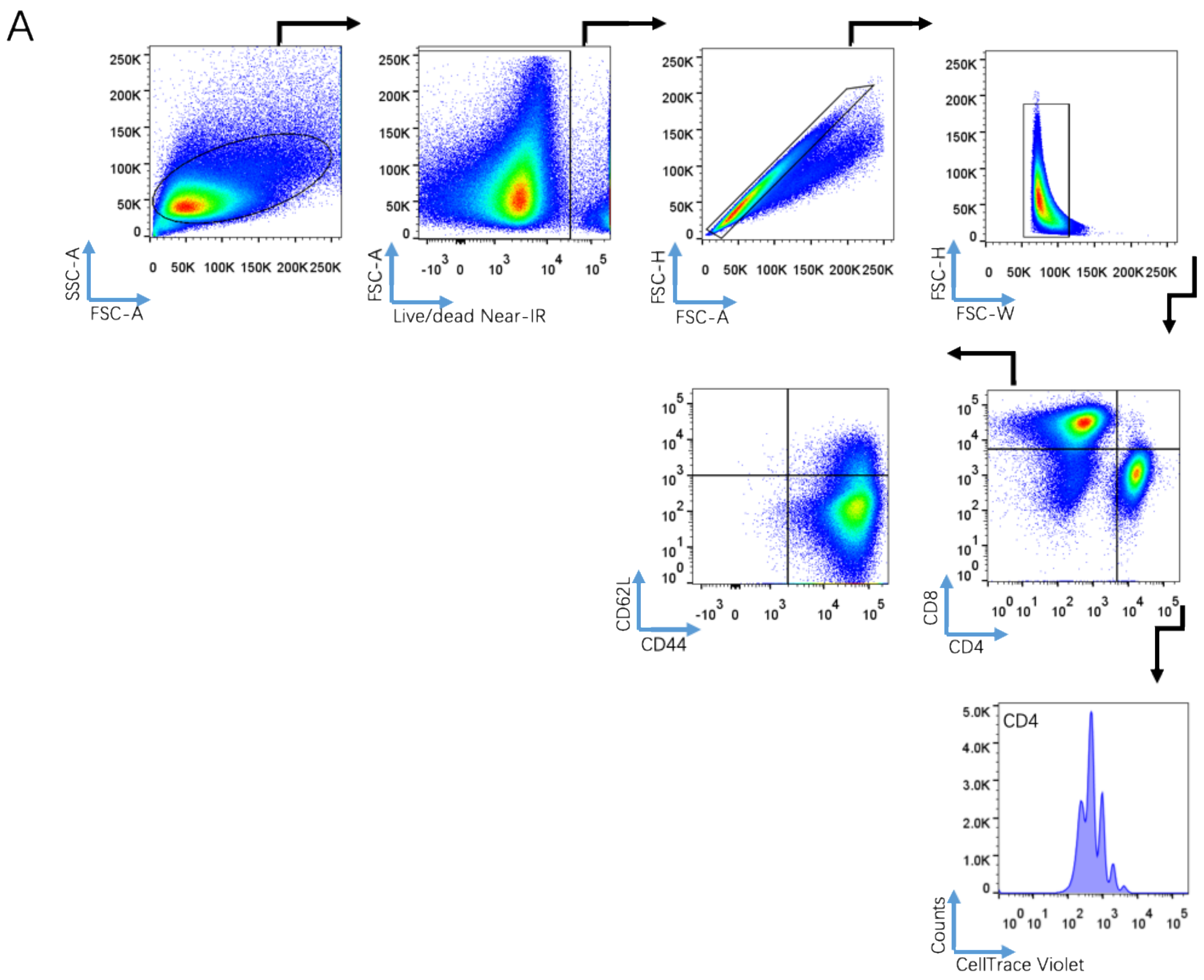

Supplementary Figure S18B. Representative flow gating strategy for intracellular IFN γ staining.

Activated T cells was initially gated with SSC-A & FSC-A, then doublet exclusion and live cells were gated. The CD8 T cells were the gated for intracellular IFN γ production.

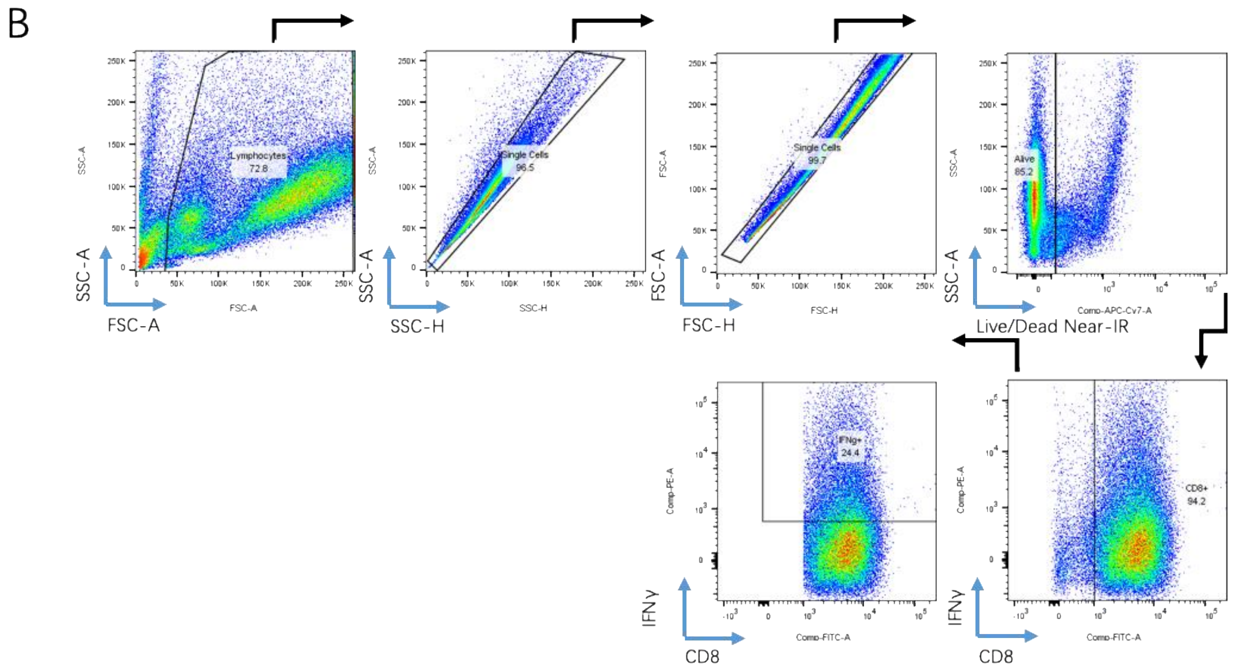

Supplementary Figure S18C. Representative gating strategy for staining of isolated lymph nodes.

Single cell suspensions of lymph node lymphocytes were initially gated with SSC-A & FSC-A, then doublet exclusion and live cells were gated. The CD45+ cells were then gated for CD11b, CD19, and CD3 single positive populations. CD3+ cells were gated for CD8 and CD4 populations.

.

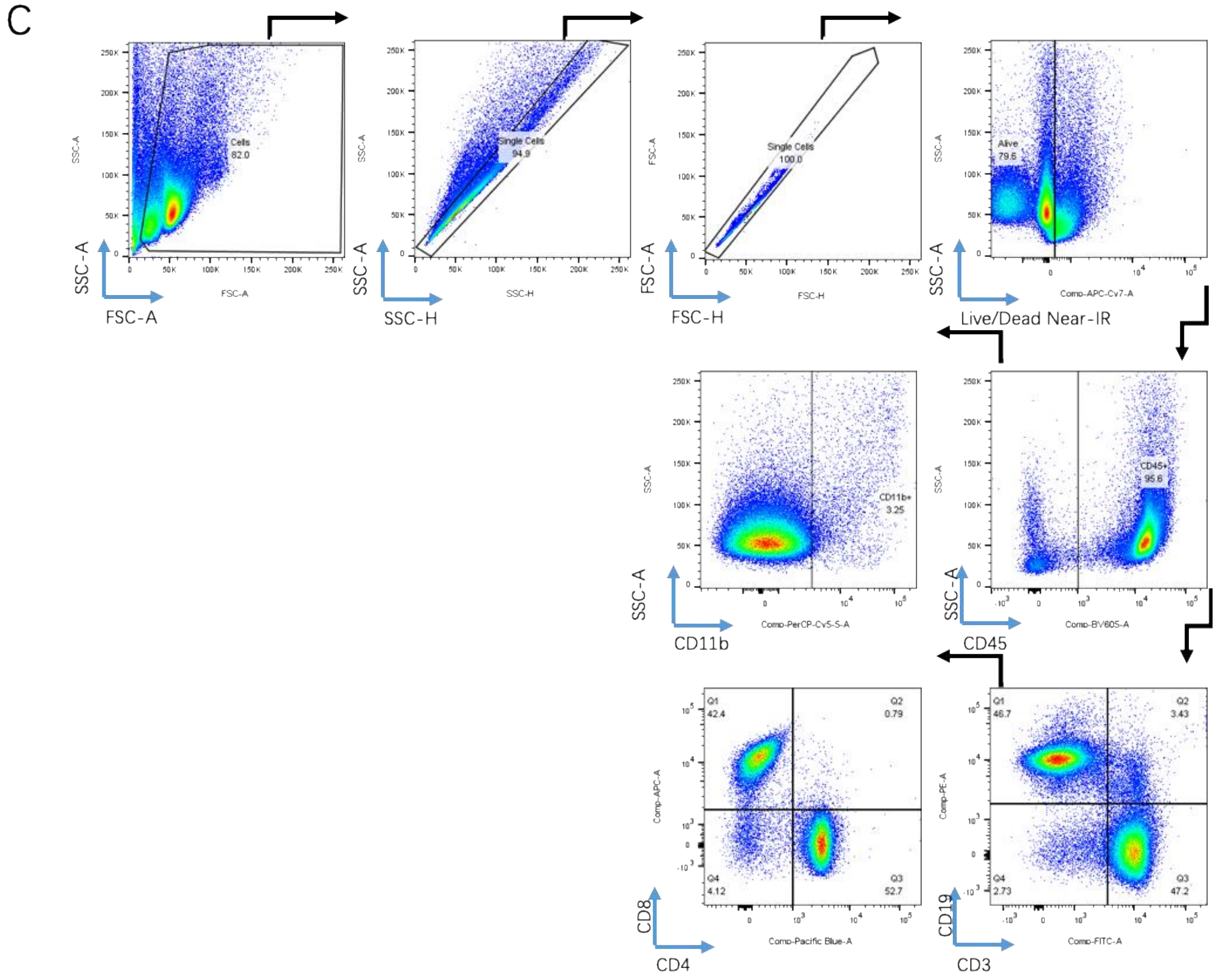

Supplementary Figure S18D. Representative gating strategy for staining of spleen and lymph node depletion studies. Single cell suspensions of spleen and lymph node lymphocytes were initially gated with SSC-A & FSC-A, then doublet exclusion and live cells were gated. The CD3+ cells were then gated for and CD4 and CD8 populations were gated off of CD3+ cells.

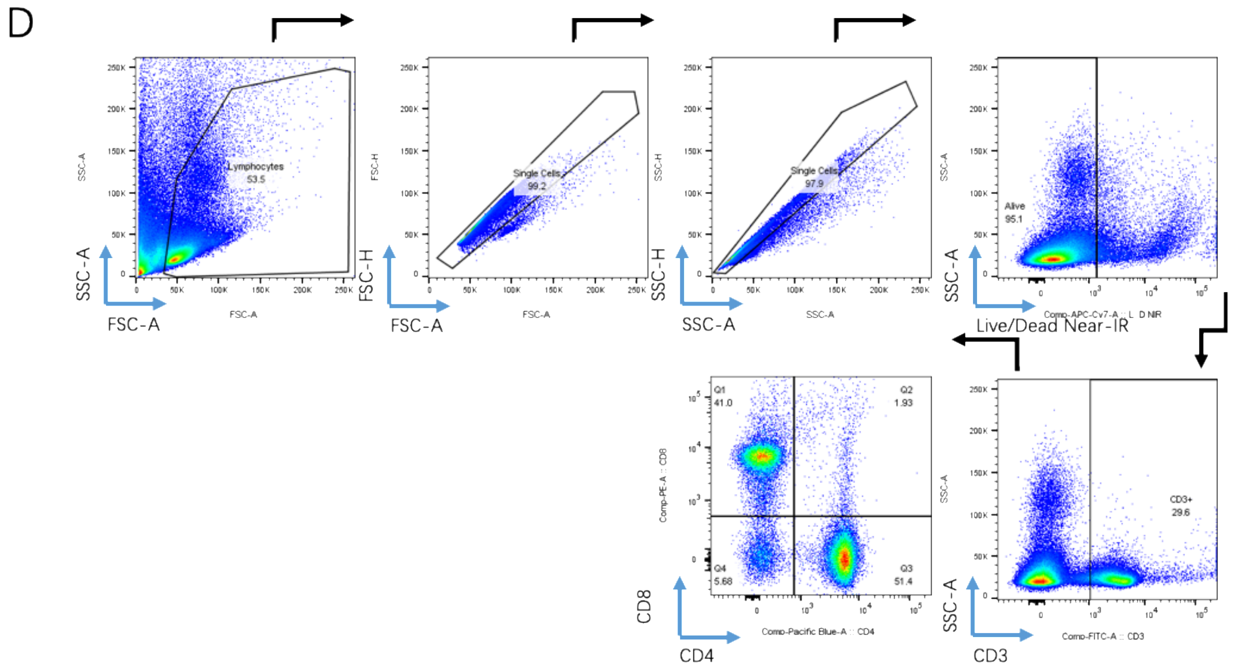

Supplement: Supplementary file 2 — Supplementary Information [file 41467_2020_17756_MOESM2_ESM.pdf]
