# Supplementary material for: T-cells produce acidic niches in lymph nodes to suppress their own effector functions
Source: Nat Commun. 2020 Aug 17;11:4113. doi: 10.1038/s41467-020-17756-7 (PMC7431837; doi:10.1038/s41467-020-17756-7)
Supplement: Supplementary file 3 — Reporting Summary [file 41467_2020_17756_MOESM3_ESM.pdf]

nature research

Corresponding author(s): Gillies, Robert J.

Last updated by author(s): Jun 16, 2020

Reporting Summary

Nature Research wishes to improve the reproducibility of the work that we publish. This form provides structure for consistency and transparency in reporting. For further information on Nature Research policies, see Authors & Referees and the Editorial Policy Checklist .

Statistics

For all statistical analyses, confirm that the following items are present in the figure legend, table legend, main text, or Methods section.

n/a	Confirmed	
☐	☒	The exact sample size (*n*) for each experimental group/condition, given as a discrete number and unit of measurement
☐	☒	A statement on whether measurements were taken from distinct samples or whether the same sample was measured repeatedly
☐	☒	The statistical test(s) used AND whether they are one- or two-sided<br>*Only common tests should be described solely by name; describe more complex techniques in the Methods section.*
☒	☐	A description of all covariates tested
☒	☐	A description of any assumptions or corrections, such as tests of normality and adjustment for multiple comparisons
☐	☒	A full description of the statistical parameters including central tendency (e.g. means) or other basic estimates (e.g. regression coefficient) AND variation (e.g. standard deviation) or associated estimates of uncertainty (e.g. confidence intervals)
☐	☒	For null hypothesis testing, the test statistic (e.g. *F*, *t*, *r*) with confidence intervals, effect sizes, degrees of freedom and *P* value noted<br>*Give P values as exact values whenever suitable.*
☒	☐	For Bayesian analysis, information on the choice of priors and Markov chain Monte Carlo settings
☒	☐	For hierarchical and complex designs, identification of the appropriate level for tests and full reporting of outcomes
☒	☐	Estimates of effect sizes (e.g. Cohen's *d*, Pearson's *r*), indicating how they were calculated

Our web collection on statistics for biologists contains articles on many of the points above.

Software and code

Policy information about availability of computer code

Data collection	Data were collected on the described instruments using the built in code
Data analysis	Data were analyzed using Excel, Prism, and Sigma Plot on both Windows and Mac platforms. Multiple versions were used. Custom code was written in MatLab for image processing of data in figures 1 G,H,I, and for steady-state modeling of Figures 2 G,H and is available upon request to corresponding author P.S.

For manuscripts utilizing custom algorithms or software that are central to the research but not yet described in published literature, software must be made available to editors/reviewers. We strongly encourage code deposition in a community repository (e.g. GitHub). See the Nature Research guidelines for submitting code & software for further information.

Data

Policy information about availability of data

All manuscripts must include a data availability statement. This statement should provide the following information, where applicable:

- Accession codes, unique identifiers, or web links for publicly available datasets
- A list of figures that have associated raw data
- A description of any restrictions on data availability

High resolution image data are available upon request from either of the corresponding authros (PS or RJG). Data supporting all plots in Figure 1-4 are available in SOURCE DATA.

Field-specific reporting

Please select the one below that is the best fit for your research. If you are not sure, read the appropriate sections before making your selection.

Life sciences study design

All studies must disclose on these points even when the disclosure is negative.

Sample size	Power calculations were not performed. At least two (and more often 3) technical replicates were used for each Biological replicate. Biological replicates were repeated until low variance gave sufficient confidence in the statistically significant differences or similarities. All valuees for N are provided
Data exclusions	no collected data were excluded from analyses
Replication	Numbers of biological replicates were various and are all provided in figure legends
Randomization	Randomization was not relevant to this work. No animal experiments performed in this work required randomization.
Blinding	Blinding was used in generating image quantification reported in figure 1F. Blinding was not performed for other experiments.

Reporting for specific materials, systems and methods

We require information from authors about some types of materials, experimental systems and methods used in many studies. Here, indicate whether each material, system or method listed is relevant to your study. If you are not sure if a list item applies to your research, read the appropriate section before selecting a response.

Materials & experimental systems

n/a	Involved in the study	
☐	☒	Antibodies
☐	☒	Eukaryotic cell lines
☒	☐	Palaeontology
☐	☒	Animals and other organisms
☒	☐	Human research participants
☒	☐	Clinical data

Methods

n/a	Involved in the study	
☒	☐	ChIP-seq
☐	☒	Flow cytometry
☒	☐	MRI-based neuroimaging

Antibodies

Antibodies used	Antibodies used in this work are completely described in Methods, as well as Supplemental Table S3.
Validation	Multiple antibody lot numbers were used and each was validated by the flow cytometry core facility according to the manufacturer prior to used and titered for appropriate staining by us. In general, antibodies were used at a dilution of 1 ul per 100 ul staining buffer per 106 cells.

Eukaryotic cell lines

Policy information about cell lines

Cell line source(s)	Jurkat cell was purchased from ATCC.
Authentication	no authetication was performed as cells were used immediately upon receipt.
Mycoplasma contamination	cells tested negative for mycoplasma
Commonly misidentified lines (See ICLAC register)	No commonly mis-identified cell lines were used in this study

Animals and other organisms

Policy information about studies involving animals; ARRIVE guidelines recommended for reporting animal research

Laboratory animals	Female B6 (C57BL/6), Pmel, OT-I, OT-II and TDAG8 knockout (TDAG8 KO) mice on the C57BL/6 background were bred and housed at the Animal Research Facility of the H. Lee Moffitt Cancer Center and Research Institute (Tampa, FL). Eight-to ten-week old Balb/c, C57BL/6 and nu/nu mice (male, 22-25 g) were purchased from The Jackson Laboratory Laboratory and housed in ventilated isolette cages at 68-79 oF and 30-70% humidity with 12:12 hr light:dark cycles.
Wild animals	no wild animals were used in this study
Field-collected samples	no field collected animals were used in this study.

Ethics oversight | All animals were maintained under Institutional Animal Care and Use Committee (IACUC) at H. Lee Moffitt Cancer Center.

Note that full information on the approval of the study protocol must also be provided in the manuscript.

Flow Cytometry

Plots

Confirm that:

- [x] The axis labels state the marker and fluorochrome used (e.g. CD4-FITC).
- [x] The axis scales are clearly visible. Include numbers along axes only for bottom left plot of group (a 'group' is an analysis of identical markers).
- [x] All plots are contour plots with outliers or pseudocolor plots.
- [x] A numerical value for number of cells or percentage (with statistics) is provided.

Methodology

Sample preparation

Fresh isolated T cells were activated at pHe 7.4 or pHe 6.6 for 72 hours. Cells were collected and wash by PBS twice, then stained in FACS buffer with the following antibodies for flow cytometric analysis: CD3, CD4, CD8, CD44 and CD62L (all from BD Biosciences). Live/Dead fixable near-IR (Invitrogen) was used to exclude dead cells before analysis. To analyze intracellular marker IFNγ, cells were incubat ed with 1μL/mL GoldgiPlug (BD Bioscience) for 3 hours, stained with surface marker and Live/Dead dye, fixed and permeabilized by Fixation/Permeabilization Solution Kit (BD Biosciences), and then stained with anti-IFNγ antibody.

Instrument

Samples are analyzed by LSR II Flow Cytometer (BD Biosciences).

Software

Samples are analyzed by LSR II Flow Cytometer (BD Biosciences).

Cell population abundance

N/A

Gating strategy

Samples were analyzed by gating on cells (Fsc-A vs Ssc-A) and excluding debris. Dublets were then excluded by sequentially gating on Fsc-A vs Fsc-H and Ssc-A vs Ssc-H. Single cells were then gated for viability via Live/Dead NIR null/low. Viable, single cells were then gated based upon additional markers according to the experiment performed.

- [x] Tick this box to confirm that a figure exemplifying the gating strategy is provided in the Supplementary Information.
